# Chiglitazar diminishes the warburg effect through PPARγ/mTOR/PKM2 and increases the sensitivity of imatinib in chronic myeloid leukemia

**DOI:** 10.1186/s40164-024-00589-1

**Published:** 2024-12-18

**Authors:** Hongpeng Duan, Qian Lai, Yuelong Jiang, Liuzhen Yang, Manman Deng, Zhijuan Lin, Weihang Shan, Mengya Zhong, Jingwei Yao, Li Zhang, Bing Xu, Jie Zha

**Affiliations:** 1 Department of Hematology, The First Affiliated Hospital of Xiamen University, School of Medicine, Xiamen University, Xiamen, 361003 China; 2 Key Laboratory of Xiamen for Diagnosis and Treatment of Hematological Malignancy, No. 55, Zhenhai Road, Xiamen, 361003 Fujian People’s Republic of China

**Keywords:** Chronic myeloid leukemia, Glucose metabolism, Imatinib resistance, Chiglitazar, PPARγ

## Abstract

**Background:**

A tyrosine kinase inhibitor (TKI) such as Imatinib (IM) is the preferred treatment for Chronic Myeloid Leukemia (CML). However, the emergence of IM resistance presents a significant challenge to disease management. A characteristic of cancer cells, including IM-resistant CMLs, are characterized by heightened uptake of glucose and aberrant glycolysis in the cytosol, which is known as the Warburg effect. In addition to its potential to modulate the Warburg effect, Chiglitazar (Chi), a compound that regulates glucose metabolism, has also been investigated for its implication in cancer treatment. This suggests that combining Chi with IM may be a therapeutic strategy for overcoming IM resistance in CML.

**Methods:**

Sensitive and IM-resistance CML cells were treated with Chi in vitro, followed by detecting of extracellular acidification rate (ECAR) using a Seahorse XF Analyzer. CML cell proliferation, cell cycle distribution, and apoptosis were tested by CCK-8 assay and flow cytometry. RNA sequencing was utilized to investigate potential transcriptional changes induced by Chi usage. In vivo studies were conducted on immunodeficient mice implanted with CML cells and given Chi and/or IM later. Tumor growth was monitored, as well as tumor burden and survival rates between groups.

**Results:**

Our metabonomic, transcriptomic, and molecular biology studies demonstrated that Chi, in part, diminished the Warburg effect by reducing glucose and lactate production in imatinib-resistant CML cells through the PPARγ/mTOR/PKM2 pathway. This modulation of glucose metabolism resulted in reduced cell proliferation and enhanced sensitivity to IM in imatinib-resistant CML cells in vitro. Rescue assay by introducing shPPARγ or mTOR activator verified the underlying regulatory pathway. Also, the combination of Chi and IM synergistically increased the sensitivity of IM in vivo and prolonged the survival of imatinib-resistance CML transplanted mice.

**Conclusions:**

Our results demonstrated the potential of Chi to overcome IM resistance in vitro and in vivo. By inhibiting the Warburg effect through the PPARγ/mTOR/PKM2 pathway, Chi resensitizes CML cells towards imatinib treatment. Combining IM with Chi is an alternative therapeutic option for CML management, especially for IM-resistant CML patients.

**Supplementary Information:**

The online version contains supplementary material available at 10.1186/s40164-024-00589-1.

## Background

CML is an aggressive myeloproliferative disorder caused by the translocation of chromosomes 9 and 22 within hematopoietic stem cells [1]. This event elicits the expression of the aberrantly activated BCR::ABL protein, which governs the dysfunction of key intracellular signaling pathways/proteins essential for the survival and proliferation of hematopoietic progenitor cells [2, 3]. Despite the transformative impact of tyrosine kinase inhibitors (TKIs) such as imatinib (IM), which target BCR::ABL fusion oncoproteins harbored in a significant proportion of patients (25% initially and up to 90% in TKI-resistant cases) [4–6]. Numerous investigations have revealed cellular processes linked to drug resistance, including the activation of key signaling pathways, cell cycle disruptions, abnormal epigenetic events, aberrant stem cell niche microenvironments, and metabolic shifts [7–9]. The clinical use of TKIs like imatinib, nilotinib, and dasatinib has substantially extended the overall survival of CML patients [10–13]. However, long time TKI therapy is ultimately associated with drug toxicity, acquired resistance, and significant economic burden [14, 15]. Furthermore, about half of the patients experience rapid relapse upon treatment cessation [16]. Thus, it is evident that new therapeutic strategies based on the biological characteristics of TKI-resistant CML cells are urgently needed.

As evidenced by stable isotope-assisted metabolomics, primitive CML cells rely heavily on oxidative metabolism [8, 17]. Metabolic reprogramming offers a promising therapeutic alternative for CML treatment. Recent studies have demonstrated that the peroxisome proliferator-activated receptor (PPAR), specifically its α, β/δ, and γ subtypes, exerts a profound influence on the Warburg effect and metabolic state of tumor cells [18, 19]. This influence is manifested through various mechanisms, including the direct regulation of pivotal metabolic enzymes such as phosphofructokinase (PFK) and citric acid cycle enzymes. PPAR also elicits its effects through inhibiting mTOR signaling pathway and suppressing HIF-1α and Wnt expression activity [20–22]. In addition, PPAR ligands also possess anticancer properties against various cancers such as colon, pancreatic, breast, prostate, and leukemia [23, 24]. It has recently been discovered that thiazolidinediones (TZDs), a class of antidiabetic drugs, are undergoing clinical trials for the treatment of hematopoietic malignancies [24, 25]. It is important to note that TZDs’ side effects have limited their clinical applications in cancer treatment [26]. Our previous study demonstrated that Chi exerted its antileukemic effects by affecting stem cell survival through the PPARα/HIF1α/PGK1 pathway [27]. As of yet, little is known about Chi’s metabolic regulation and potential antileukemic effects on IM-resistant CML cells.

This study aimed to explore the potential of glucose metabolism modulation as a strategy to resensitize CML cells to IM therapy by employing Chi to influence glycolysis. Our findings demonstrated the synergistic effect of Chi and IM in inhibiting the CML cell proliferation. We showed that Chi treatments significantly reduce extracellular acidification rates (ECAR) and cellular ATP production in CML cells. As a result of these energy metabolic modulations, CML cells were induced by combined therapy to undergo apoptosis. Our study suggests the potential of combing Chi and IM to overcome IM resistance in CML cells.

## Methods

### Reagents and cell culture

IM was purchased from MedChemExpress LLC (Shanghai, China) and dissolved in dimethyl sulfoxide (DMSO) (Invitrogen Corp., Waltham, MA, USA) to yield a 10 mM stock solution. Chi, obtained from Chipscreen Biosciences Co., Ltd. (Shenzhen, Guangdong, China), was dissolved in DMSO to obtain a 100 mM stock solution. MHY1458 was purchased from Selleck Inc. (Shanghai, China) dissolved in DMSO to obtain a 10 mM stock solution. Subsequently, it was diluted to specified concentrations in culture media for other experiments. The CML cell lines K562 and Ba/F3 (BCR::ABL-T315I) were kindly provided by Professor Liu XL and Professor Jiang Q (Peking University People’s Hospital) in Beijing, China. K562 and Ba/F3 cells were cultured in RPMI-1640 (Hyclone, Thermo Fisher Scientific, Logan, USA) supplemented with 10% fetal bovine serum (Gibco, Life Technologies, NY, USA), 100 units/mL penicillin, and 100 μg/mL streptomycin at 37 °C in a 5% CO_2_ incubator. K562 IM-resistant cell line (K562R) was cultured in imatinib at gradually increased concentrations and cultured in a supplemented medium containing 1 μM imatinib mesylate to acquire drug resistance. IM and simvastatin (SIM) were obtained from MedChemExpress (Shanghai, China) and prepared accordingly.

### Cell proliferation assay

The cell-killing effects of IM and Chi were evaluated using the Cell Counting Kit-8 (Shandong Sparkjade Biotechnology Co., Ltd, China). Cells were seeded in 96-well plates at a density of 4 × 10^4^ cells per well and were treated with designated doses of IM or Chi, either alone or in combination, for 48 h. Following the addition of the CCK-8 reagent, absorbance was measured at 450 nm using a microplate reader (ELx800; BioTek Instruments Inc, Winooski, VT). All experiments were performed in triplicate and repeated three times. The half-maximal inhibitory concentration (IC50) was calculated using SPSS 20.0 (SPSS Inc, Chicago, IL, USA). The combination index (CI) was computed using the Chou-Talalay method with Calcusyn software, which was used to quantify therapeutic synergy based on cell viability data (CI < 1 indicates synergy).

### Cell apoptosis and cell cycle assay

For apoptosis evaluation, cells were subjected to treatment with either Chi or IM, independently or in combination, for 48 h. Subsequently, they were stained following the manufacturer’s guidelines using Annexin-V-FITC/PI (eBioscience, San Diego, CA, USA). The proportion of Annexin-V positive (apoptotic) cells was ascertained through flow cytometry (FACSCalibur, BD Biosciences) and data were processed using FlowJo software. In parallel, cell cycle analysis was performed on similarly treated cells, which were fixed in ethanol, stained with PI/RNase, and examined via flow cytometry. The resulting cell cycle distribution was scrutinized using flow cytometry and FlowJo software.

### Gene knockdown and expression assay

We designed the shPPAR**γ** sequence (GGAGAACAATCAGATTGAAGC) and cloned into lentiviral vector ordered from Genechem (Shanghai, China). The shPPARγ plsmid was transfected into K562R cells using lipofectamine 2000 transfectiong kit (ivitrogen) 24 h after Chi treatment following the protocol. For apoptosis assay under PPARγ knockdown condition, we transfected the plasmid and cultured the K562R cell with the drug containing medium for 48 h at the same day and then processed the cells to FACS assay.

### RNA sequencing and data analysis

Total RNA samples from different conditions were subjected to RNA sequencing (RNA-seq) on Illumina Hiseq 2500 platform. Released sequencing data were trimmed and quality filtered using cutadapt (http://cutadapt.readthedocs.io, version 1.2.0). Alignment was performed using Hisat2 with human (hg38) genome as a reference [28]. Quantified transcripts were generated by Feature Count program [29]. They then subjected to differential expression analysis using Deseq2 program [30]. The Venn diagram was generated using the ggVenn program. GO analysis was done using Database for Annotation, Visualization and Integrated Discovery (DAVID) [31, 32]. GSEA analysis was carried out to verify the correlation between differential expressed genes and biological states.

### Glucose and lactic acid measurement

For glucose uptake analysis, 2 × 10^6^ K562 or K562R cells were seeded into a 24-well plate and treated with imatinib and/or siglitin sodium for 24 h. A glucose uptake assay kit (A154-1-1, Nanjing Jiancheng Bioengineering Institute, China) was used following the manufacturer’s instructions. The glucose levels in the culture medium were compared before and after the 24-h treatment, and absorbance at 488 nm was measured and normalized to the cell number. This comparison allowed for the assessment of glucose uptake among the different groups. For lactic acid analysis, 2 × 10^6^ K562 or K562R cells were seeded into a 24-well plate and incubated at 37 °C for 24 h. Following treatment with imatinib and/or siglitin sodium for 24 h, the cells were lysed to collect intracellular lactic acid. A lactate assay kit (BC2235, Solarbio, China) was used per the manufacturer’s instructions. Absorbance at 450 nm was recorded.

### Metabolic profiling of CML cells

Glycolysis levels in K562 or K562R cells were gauged using lactate production (BC2235, Solarbio, China) and glucose uptake assays (A154-1-1, Nanjing Jiancheng Bioengineering Institute, China), following the respective assay kits’ instructions. Concurrently, real-time analysis of cellular metabolism was conducted on XFe96 extracellular flux analyzer (Seahorse Bioscience, North Billerica, MA, USA). Cell plates were pre-coated with poly-lysine (Pernoside, Wuhan, China) to facilitate wall attachment of K562 and K562R cells. 1 h before the assay, changed the culture medium to pre-warmed hippocampal basal medium supplemented with 1 mM pyruvate, 2 mM glutamine, and 10 mM glucose. Cells then were incubated in a CO_2_-free incubator at 37 °C for 1 h. Glucose (10 mM), oligomycin (2 μM), and 2-Deoxy-d-glucose (2-DG, 100 mM) were injected into ports A, B, and C, respectively, for ECAR analysis. Results were normalized based on the cell numbers.

### Western blot analysis

Whole cell lysates (30 μg/lane) of each sample went through 10% or 12.5% SDS-PAGE gel and subsequently transferred to a PVDF membrane (Millipore Corp., Burlington, MA, USA). After blocking non-specific binding, PVDF membranes were incubated at 4 °C overnight with primary antibodies (PARP, rabbit monoclonal, 1:1000; Caspase3, rabbit monoclonal, 1:1000; GAPDH, rabbit monoclonal, 1:1000; STAT5, rabbit monoclonal, 1:1000; P-STAT5, rabbit monoclonal, 1:1000; PPARγ, rabbit monoclonal, 1:1000; MTOR, rabbit monoclonal, 1:1000; PKM2, mouse monoclonal, 1:1000; Cell Signaling Technology, Inc., Danvers, MA, USA). PVDF membranes were then incubated with horseradish peroxidase-conjugated secondary antibodies for 1 h at room temperature. Immunoreactive bands were detected using ECL buffer (Aase Bio, Shanghai, China).

### Chronic myeloid leukemia cell derived xenograft model

NOD-Prkdc−/−IL2rg−/− (NOD/SCID) mice (6 weeks old) obtained from the Animal Care Institute of Xiamen University were raised in a pathogen-free environment. To establish a Cell Derived Xenograft (CDX) model, mice received 1.5 Gy sublethal irradiation followed by tail vein injection of 1 × 10^6^ K562R cells and were randomly divided into 4 groups (n = 14). On day 7, peripheral venous blood samples were taken, and all groups of mice were euthanized for further analysis of human CD45 expression in the spleen (Spleen tissue human CD45 > 1%, 7 days post-transplantation). 0 mice were administered oral gavage carrier (methylcellulose/tween-80), imatinib (50 mg/kg/day), chiglitazar (15 mg/kg/day), or their combination for 12 days to determine drug efficacy. The percentage of human CD45 + CML cell infiltration in bone marrow and spleen leukocytes was measured to determine the drug response in all experimental groups of mice. For Ki67 immunohistochemistry (IHC) and hematoxylin and eosin (H&E) staining, tissue sections were deparaffinized, rehydrated, and antigen-retrieved. Ki67 primary antibody (TA800648, ZSGB-Bio) was applied overnight at 4 °C. After washing, a secondary antibody and 3,3′-Diaminobenzidine (DAB) chromogen were used for signal detection. Counterstaining was done with hematoxylin. Animals were used in accordance with a protocol approved by the Laboratory Animal Center of Xiamen University.

### Statistical analysis

Data were presented as mean ± standard deviation (SD) from three independent experiments and analyzed using GraphPad Prism 6 software (GraphPad Software, San Diego, CA, USA). Statistical comparisons between two groups were conducted using Student’s t-tests (unpaired, two-tailed) or one-way analysis of variance (ANOVA) followed by Tukey’s post-hoc tests. P < 0.05 was considered statistically significant. All data generated and analyzed during this study are available upon request from the corresponding author.

## Results

### IM-resistant cells showed a increased glucose uptake and lactate production

**Fig. 1:**
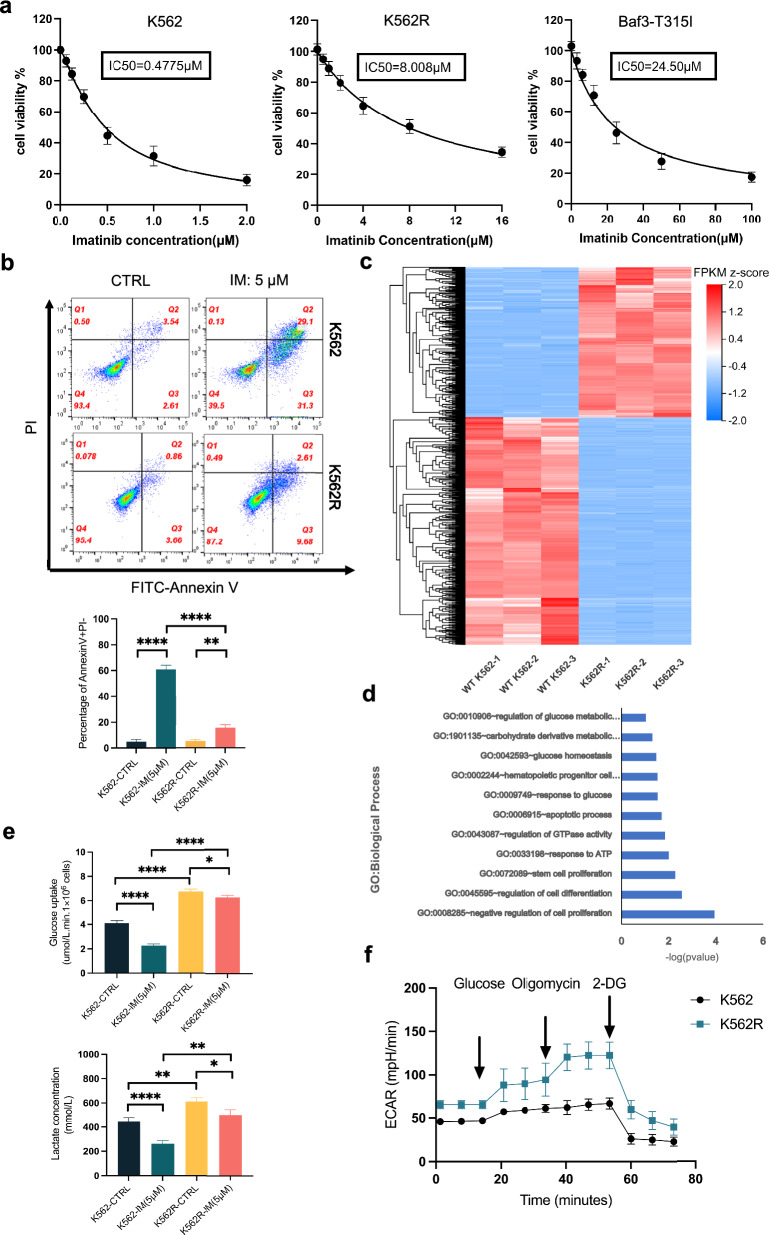
IM-resistant cells showed a increased glucose uptake and lactate production. **a** Cell viability percentage of K562, K562R and Baf3-T315I cells after 48 h of imatinib treatment. **b** Flow cytometry apoptosis assay of K562 and K562R cells following imatinib treatment. **c** Heatmap showing differentially expressed genes in K562 and K562R cells (q < 0.01 and |log fold change (FC)| > 2). **d** KEGG and GO enrichment analysis of DEGs (q < 0.01 and |log fold change (FC)| > 2). **e** Glucose uptake and lactate production in K562 and K562R cells after IM treatment for 24 h. **f** For the glycolysis analysis, cells were firstly seeded in plate for 6 h, and then cells were sequentially treated with glucose (10 mM), oligomycin (2 μM), 2-DG (100 mM). Data are presented as mean ± SD. NS < 0.1234, *P < 0.03, **P < 0.0021, ***P < 0.0002, ****P < 0.0001

We first sought to evaluate the cellular responses of IM-resistant CML to IM treatment. Therefore, human and mice IM-resistant CML cell lines K562R (drug induced) and Baf3-T315I (T315I mutation) were exposed to 48 h IM treatment in this study [33]. Cell viability assays were performed to assess the proliferative abilities of cell lines when treated with IM. The IC50 values of IM for K562, K562R, and Baf3 cells were 0.48 μM, 8.01 μM, and 24.37 μM (Fig. 1a). Then we assessed cell line apoptosis using Annexin V-FITC/PI staining. The FACS analysis revealed significantly lower apoptosis percentage in K562R (Fig. 1b, Additional file 1: Fig. S1a–c). We chose 5 μM as IM’s concentration to conduct further tests which is between the IC50 values of IM for K562 and K562R (Fig. 1a). To explore the underlying transcriptional mechanism of IM resistance, RNA sequencing was performed on K562 and K562R cell lines. Differential expression analysis revealed 967 downregulated and 636 upregulated genes (Fig. 1c, Additional file 2: Table S1). Further, KEGG and GO enrichment analyses of differential expressed genes (DEGs) highlighted significant alterations in pathways associated with glycolysis and oxidative phosphorylation (Fig. 1d, Additional file 3: Table S2). These findings reinforce previous research suggesting that CML-resistant cells rely on enhanced oxidative metabolism for survival, albeit without a fully elucidated mechanism [34]. So, we assessed the glucose uptake and lactate production in K562 and K562R cells by measuring the glucose and lactate levels in the culture medium. Interestingly, K562R cells displayed enhanced glucose uptake and lactate production, indicating a metabolic shift towards glycolysis, a phenomenon known as the Warburg effect (Fig. 1e). To test the Warburg effect in IM-resistant cells, we detected the Oxygen Consumption Rate (OCR) and Extracellular Acidification Rate (ECAR) level. IM-resistant cells showed higher ECAR levels and lower OCR levels than IM-sensitive cells (Fig. 1f, Additional file 1: Fig. S1d). Our results confirmed that IM-resistant cells’ metabolic program depends more on glycolysis.

### Chiglitazar diminished the glucose uptake in IM-resistant cells

**Fig. 2:**
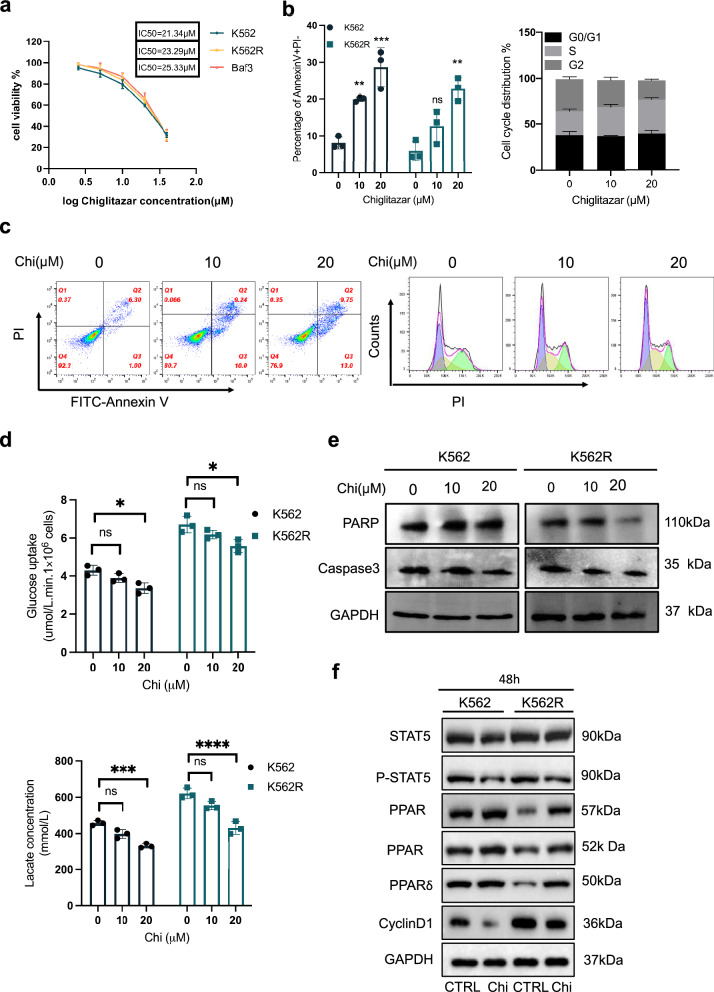
Chiglitazar diminishes the Warburg effect in imatinib-resistant K562R cells. **a** Cell viability percentage of K562R cells after 48 h of Chiglitazar treatment. **b** Induction of apoptosis by 48 h of treatment with Chiglitazar, and cell cycle distribution upon 24 h Chiglitazar treatment in K562R cells. **c** Typical representative diagrams of apoptosis and cell cycle. **d** Glucose uptake and lactate production in K562R cells treated with Chiglitazar for 48 h. **e** Western blotting analysis of PARP and Caspase-3 before and after Chi treatment for 48 h. **f** Western blotting analysis of PPAR protein family, STAT5, and CyclinD1 expression levels after Chi (20 μM) treatment for 48 h. GAPDH was used as a loading control. Data are presented as mean ± SD. *NS < 0.1234, *P < 0.03, **P < 0.0021, ***P < 0.0002, ****P < 0.0001

Chiglitazar, a common antidiabetic drug, has previously shown its therapeutic effect in the treatment of acute myeloid leukemia [27]. Chi reducing glucose metabolism in leukemic stem-like cells inspired us to investigate its potential application in the treatment of CML. We first used the CCK-8 assay to evaluate the cell-killing ability of Chi in IM-sensitive cell lines (K562) and resistant cell lines (K562R and Baf3-T315I). Chi exhibited dose-dependent cytotoxicity in K562R cells, and there was no significant difference between sensitive and resistant cell lines with IC50 values of 21.34 μM (K562), 23.29 μM (K562R), and 25.33 μM (Baf3-T315I), respectively (Fig. 2a). Next, we focus on evaluating the apoptosis and cell cycle progression of K562R cell with Chi treatment. Chi did not significantly induce apoptosis in K562R cells even after 48 h of treatment, with the apoptosis rate still below 30% (Fig. 2b, c). Cell cycle distribution assay was conducted after treated with varying concentrations of Chi (0, 10, 20 μM) for 24 h and found that higher dose Chi treatment (20 μM) but not lower dose (10 μM) caused cell cycle arrest at the S phase which is consistant with our apoptotic assay (Fig. 2b, c). In addition, we investigated the influence of Chi on glucose uptake and lactate production in K562 and K562R cells. The results showed a significant decrease in glucose uptake and lactate production in each cell type after Chi treatment (Fig. 2d). We confirmed that IM-resistant cells had a higher level of glucose uptake and lactate production (Fig. 1e). Further, Chi significantly reduced resistant cells’ glucose uptake and lactate production to a level comparable to that of sensitive cells, indicating that Chi significantly inhibited the Warburg effect in resistant cells (Fig. 2d). Finally, we evaluated whether Chi would affect the expression of apoptosis-related proteins PARP and Caspase3, and the results showed a limited effect of Chi on their expression, which was consistent with our flow cytometry results (Fig. 2b, e). We also evaluated the impact of Chi on the expression of its target proteins: PPARα/γ/δ, leukemia stemness-maintaining STAT5, and cell cycle-related protein CyclinD1. The results showed that Chi could significantly inhibit the levels of P-STAT5 and reverse the downregulation of PPAR family proteins and the upregulation of CyclinD1 in resistant cell lines (Fig. 2f). Taken together, our results suggested that Chi significantly reduced the Warburg effect in IM-resistant CML cells.

### Chiglitazar resensitized imatinib-resistant CML cells

**Fig. 3:**
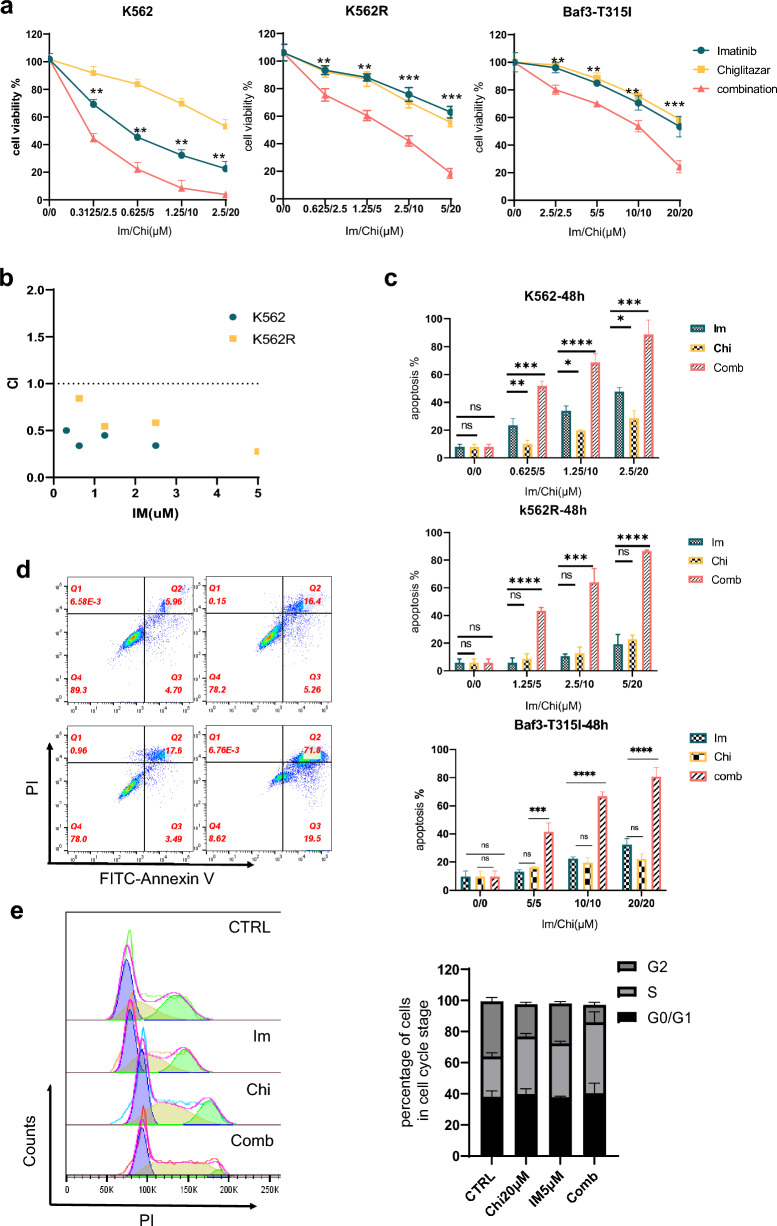
Chiglitazar resensitized Imatinib-resistant CML cells. **a** Cell viability percentage of Chi-only (20 μM), IM-only (5 μM), and their combination for 48 h in K562, K562R, and Baf3-T315I cells. **b** Combination index (CI) of Chi and IM in the indicated two cell line cells was analyzed by CalcuSyn software. CI > 1 indicates antagonist effect; CI = 1 indicates additive effect; CI < 1 indicates synergistic effect. **c** Apoptosis rate in three indicated cell lines treated with Chi-only (20 μM), IM-only (5 μM), and their combination were analyzed using flow cytometry. **d** The representative apoptosis diagrams after 48 h of combined treatment with Chi (20 μM) and IM (5 μM). **e** The representative cell cycle distribution diagrams after 24 h of combined treatment with Chi (20 μM) and IM (5 μM). Data are presented as mean ± SD. NS < 0.1234, *P < 0.03, **P < 0.0021, ***P < 0.0002, ****P < 0.0001

**Fig. 4:**
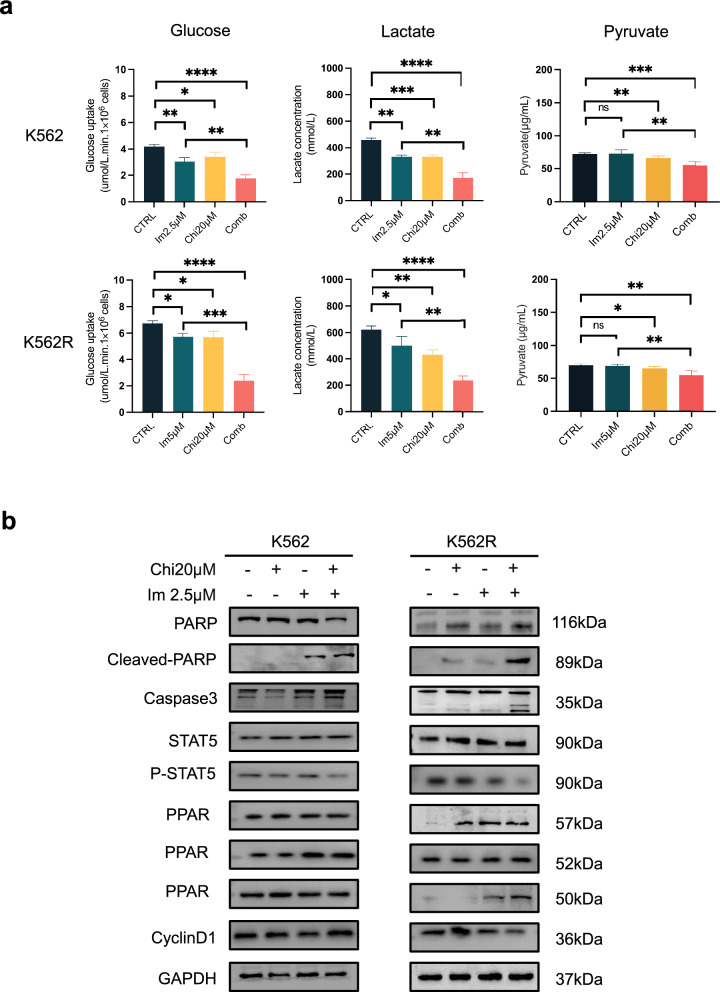
Chiglitazar reprogrom the metabolism of Imatinib-resistant CML cells. **a** Glucose uptake, lactate production, and pyruvate production in K562 and K562R cells after Chi-only (20 μM), IM-only (5 μM), and their combination for 24 h. **b** Western blotting analysis of full-length/cleaved PARP, Caspase-3, STAT5, PPAR protein family, and CyclinD1 expression levels after Chi-only (20 μM), IM-only (5 μM), and their combination treatment for 48 h in K562 and K562R cells. GAPDH was used as a loading control. Data are presented as mean ± SD. NS < 0.1234, *P < 0.03, **P < 0.0021, ***P < 0.0002, ****P < 0.0001

To evaluate the therapeutic potential of Chi in CML, we conducted a CCK8 assay to evaluate the cell viability with different concentrations of Chi and IM. As previously shown, the cytotoxic effects of either Chi or IM alone on IM-resistant cells were limited (Figs. 1, 2). The combination of different concentrations of Chi and IM at a fixed ratio (4:1) significantly decreased cell viability in both sensitive and IM-resistant cell lines (Fig. 3a). The combination index (CI) in both sensitive and IM-resistant cell lines was less than 0.85 (Fig. 3b). The sensitive cell line already displayed significant apoptosis upon IM treatment, which was significantly enhanced by the addition of Chi (Fig. 3c). Higher concentrations of IM had limited apoptosis effects in drug-resistant and T315I mutation cell lines, but were greatly enhanced by co-treatment with Chi (Fig. 3c, d). As shown in Fig. 3e, the combination of Chi and IM not only affected cell apoptosis but also the cell cycle, with cell cycle arrest observed in S phase (Fig. 3e). Glucose, lactate, and pyruvate generation were significantly decreased in cells treated with combined Chi and IM (Fig. 4a, Additional file 1: Fig. S1e). Western blot revealed upregulation in the expression of metabolism-associated gene PPARγ and downregulation of the expression of P-STAT5 (Fig. 4b). Thus, combining Chi and IM significantly induced the apoptosis of IM-resistant cell lines.

### Chiglitazar resensitized K562R cells through PPARγ/mTOR/PKM2 pathway

**Fig. 5:**
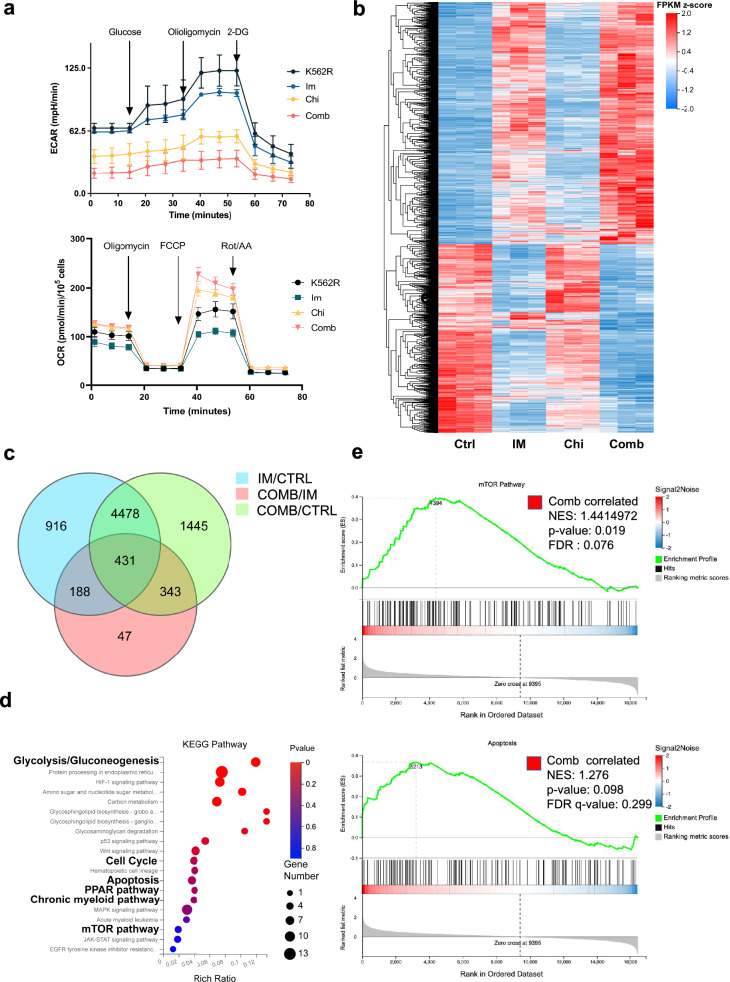
RNA-Seq identifies a potential pathway by which Chiglitazar resensitized K562R cells. **a** ECAR and OCR levels in different conditions using the Seahorse Bioscience Extra Cellular Flux Analyzer. For the glycolysis analysis, cells were firstly treated with Chi (20 μM), IM (2.5 μM for K562 cells and 5 μM for K562R cells) and their combination for 6 h, and then cells were sequentially treated with glucose (10 mM), oligomycin (2 μM), 2-DG (100 mM). **b** Heatmap showing DEGs in K562R cells treated with Chi (20 μM), IM (5 μM) and their combination for 48 h (q < 0.05 and |log fold change (FC)| > 0.5). **c** Venn diagram of different DEG groups (q < 0.05 and |log fold change (FC)| > 0.5). **d** KEGG pathway enrichment analysis of 431 overlapping DEGs. **e** GSEA analysis of mTOR pathway and apoptosis comparing combination therapy over IM-only treatment. Data are presented as mean ± SD. NS < 0.1234, *P < 0.03, **P < 0.0021, ***P < 0.0002, ****P < 0.0001

**Fig. 6:**
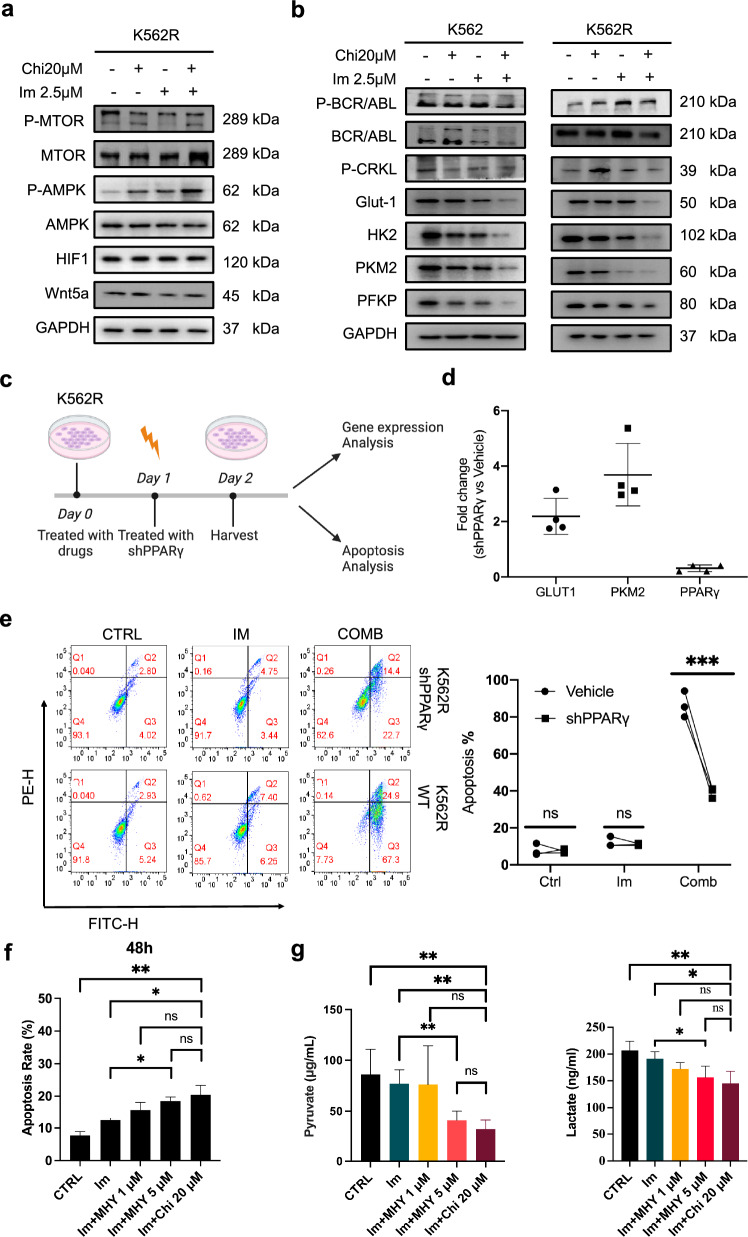
Chiglitazar resensitized K562R cells through PPARγ/mTOR/PKM2 pathway. **a** Western blotting analysis of mTOR, p-mTOR, AMPK, p-AMPK, HIF1, and Wnt5a expression levels after Chi-only (20 μM), IM-only (5 μM), and their combination treatment for 48 h in K562R cells. **b** Western blotting analysis of p-BCR/ABL, BCR/ABL, P-CRKL, Glut-1, HK2, PKM2 and PFKP expression levels after Chi-only (20 μM), IM-only (5 μM), and their combination treatment for 48 h in K562 and K562R cells. GAPDH was used as a loading control. **c** Schematic diagram of shPPARγ treatment in K562R cells. **d** The log2-fold changes in gene expression are shown. **e** Induction of apoptosis in K562R cells by 48 h of treatment with Chiglitazar, and shPPARγ upon 24 h treatment. **f** Induction of apoptosis by 48 h of treatment with MHY and Chiglitazar in K562R cells. **g** Pyruvate and lactate production after MHY and Chiglitazar treatment for 48 h. Data are presented as mean ± SD. NS < 0.1234, *P < 0.03, **P < 0.0021, ***P < 0.0002, ****P < 0.0001

The metabolic reprogramming of cancer cells is characterized by the Warburg effect, which is defined by increased glucose uptake and lactate production. Studies have indicated that prolonged IM usage correlates with increased glycolysis, potentially linked to IM resistance [35, 36]. Elevated glucose uptake and lactate production in the resistant cell line suggested an aberrant metabolic profile within the glycolytic pathway (Fig. 4b). Our analysis revealed that CML-resistant cells exhibit higher ECAR levels when compared to sensitive cells (Fig. 1f). Combing Chi and IM treatment significantly reduced the IM-resiatant cells’ ECAR level (Fig. 5a). Also, IM-resistant cells’ OCR levels were restored to those similar to IM sensitive cells when treated with Chi and IM combined regimen (Fig. 5a, Additional file 1: Fig. S1d). To elucidate how Chi restores IM sensitivity in CML cells, we conducted transcriptome sequencing studies. Interestingly, RNA-Seq showed that Chi-only treatment didn’t change K562R’s transcriptome profile a lot, but combining IM with Chi promoted the transcriptional change induced by IM treatment (Fig. 5b). Our RNA-Seq result verified the synergistic effect of Chi and IM in IM-resistant cells (Fig. 3). Then we combined three groups of DEGs and listed out 431 DEGs represented the genes that changed by adding Chi into traditional IM treatment in IM-resistant cells (Fig. 5c, Additional file 4: Table S3). KEGG enrichment analysis was employed to uncover the underlying biological process, signaling pathways, and related diseases. Glycolysis, cell cycle, apoptosis, mTOR pathway, PPAR pathway, and CML disease were highly enriched (Fig. 5d, Additional file 5: Table S4). GSEA analysis of apoptosis and mTOR pathway also confirmed that they were positively correlated to combined treatment over IM-only treatment (Fig. 5e). Given the roles of AMPK, HIF1, and mTOR signaling pathways in metabolism, we investigated whether Chi and IM-induced metabolic alterations were associated with changes in these proteins. Our results demonstrated a decrease in mTOR phosphorylation coupled with an increase in AMPK phosphorylation in K562R cells following Chi and IM combined treatment (Fig. 6a). Tumor cells maintain PKM2 activation to sustain glycolytic levels, thereby evading drug-induced cell death. Examination of Glut1, HKII, PKM2, and PFKP expression levels in CML cells treated with combined Chi and IM revealed a downregulation of Glut1 and a substantial decrease in pyruvate kinase PKM2 expression in both cell types following the combined strategy (Fig. 6b). Then we performed rescue assay to verify the PPARγ/mTOR/PKM2 pathway’s regulatory function in IM resistance mechanism. We used shPPARγ to transient knockdown PPARγ in IM-resistant cells after Chi treatment for 24 h (Fig. 6c). Our qPCR and immunoblot results confirmed the downregulation of PPARγ (Fig. 6d, Additional file 1: Fig. S1f). Also, PPARγ knockdown lead to the upregulation of Glut1 and PKM2 (Fig. 6d). Then we tested the apoptosis ratio of IM-resistant cells treated with shPPARγ. Downregulation of PPARγ significantly redued the apoptosis-induction effect when IM-resistant cells treated with IM and Chi combined treatment (Fig. 6e). Also, we introduced mTOR activator MHY1458 (MHY) to test whether MHY can mimic the apoptosis-induction and metabolic-reprogram effect of Chi treatment. We chose 1 and 5 μM (lower than 72 h IC50: 20.7 μM) to avoid the cytotoxic effect of MHY (Additional file 1: Fig. S1g). mTOR activation was showed by immunoblot test after MHY treatment (Additional file 1: Fig. S1h). The apoptosis assay verified that activation of mTOR by MHY could miminc the Chi’s apoptosis-induction in IM-resistant cells (Fig. 6f, Additional file 1: Fig. S1i). After MHY treatment, the pyruvate and lactate production were significant reduced which is similar to Chi treatment (Fig. 6g). Through a combination of metabonomic, transcriptomic, and molecular biology studies, Chi was verified to resensitize IM-resistant CML cells via the PPARγ/mTOR/PKM2 pathway.

### Chiglitazar resensitizes CML cells to imatinib in vivo

**Fig. 7:**
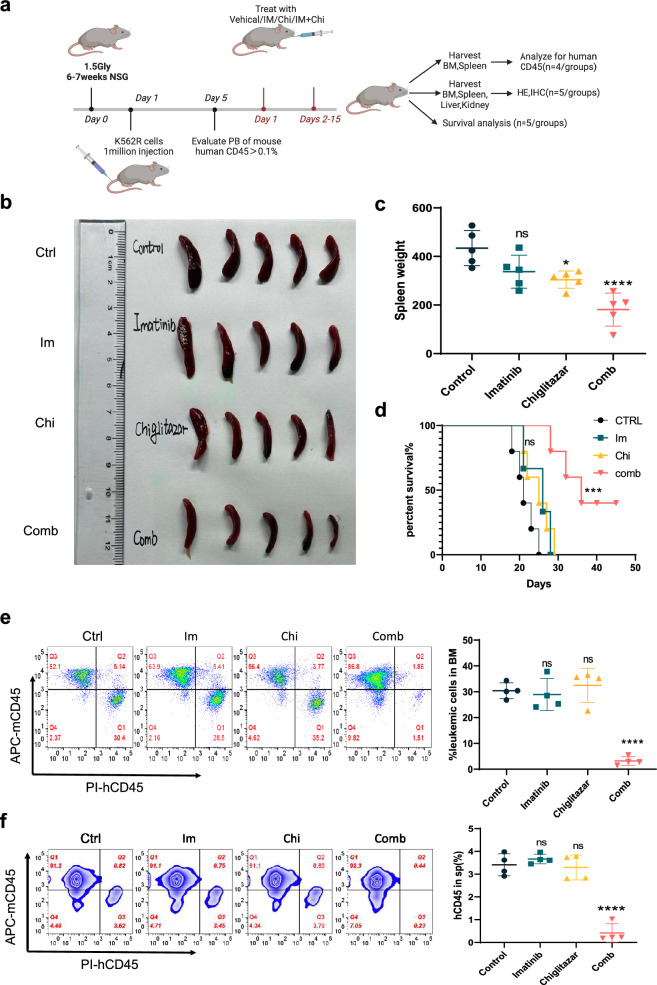
Chiglitazar resensitizes CML cells to Imatinib in vivo*.* **a** Experimental design for CDX CML mice model. **b** Representative spleen images of CDX models after 12 days of gavage carrier, IM (50 mg/kg/day), Chi (15 mg/kg/day), or their combination. **c** Spleen weight after 12 days of gavage carrier, IM (50 mg/kg/day), Chi (15 mg/kg/day), or their combination. **d** Kaplan–Meyer survival analysis of CDX models treating with gavage carrier, IM (50 mg/kg/day), Chi (15 mg/kg/day), or their combination. **e**, **f** Flow cytometry analysis showing human CD45 + CML cells in the bone marrow and spleen after 12 days of gavage carrier, IM (50 mg/kg/day), Chi (15 mg/kg/day), or their combination. Data are presented as mean ± SD. NS < 0.1234, *P < 0.03, **P < 0.0021, ***P < 0.0002, ****P < 0.0001

**Fig. 8:**
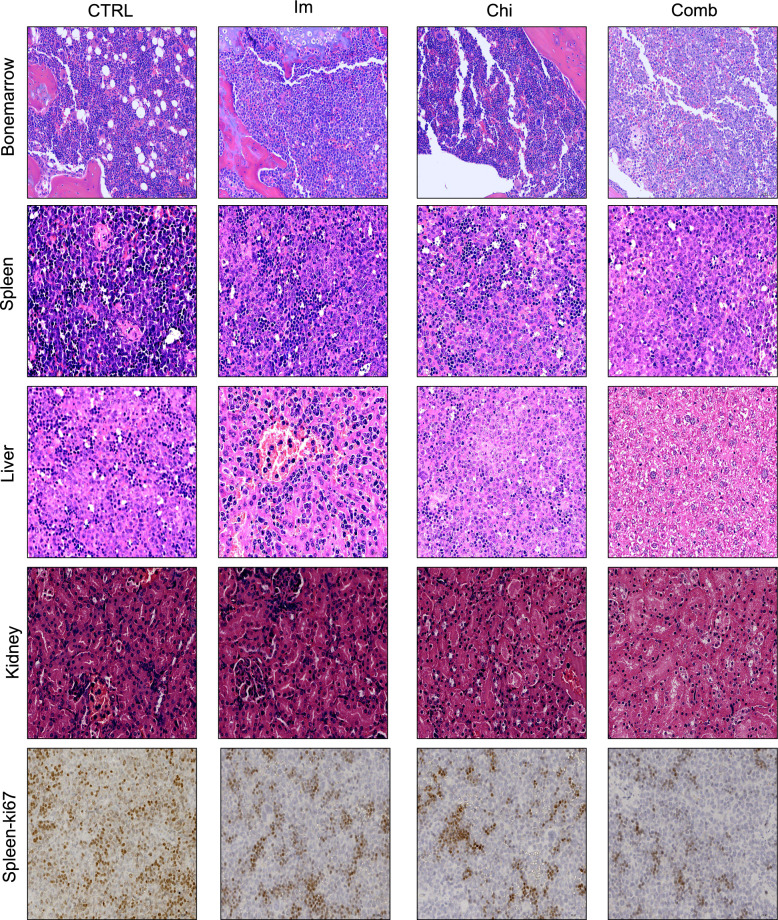
HE staining in the liver, spleen, kidneys, and bone marrow and Ki-67 staining in spleen across all treatment groups

To assess the clinical implications of our findings, we further evaluated the in vivo effects of Chi on IM-resistant CML using a K562R cell line-derived xenograft model (CDX) (Fig. 7a). Mice receiving different drug treatments showed no loss of weight throughout the experiment (Additional file 1: Fig. S1j). Upon euthanization of the mice, we noticed a significant reduction in spleen weight following combined treatment of IM and Chi compared to IM-only groups (Fig. 7b, c). Interestingly, while treated with Chi did not notably prolong the survival of CML cell-transplanted mice, combined treatment significantly extended the survival of IM-resistant mice (Fig. 7d). Flow cytometry analysis of human CD45+ cells in the bone marrow and spleen revealed a substantial reduction in human CML tumor blast following combined treatment with Chi and IM, compared to either treatment alone (Fig. 7e, f). Histological examination of the liver, spleen, kidney, and bone marrow did not reveal any significant toxicity in any of the treatment groups (Fig. 8). Furthermore, immunohistochemical analysis demonstrated a significant reduction in the proliferation marker Ki-67 in the combination treatment group compared to the other groups (Fig. 8). In summary, our in vivo data indicated that Chi, when combined with IM, significantly reduced the burden of IM-resistant CML cells and improved the IM-resistant mice models’ survival. These findings underscore the potential of this combination as a promising therapeutic strategy against IM-resistant CML.

## Discussion

TKIs have been widely used for treating CML patient that significantly improved the prognosis and life expectancy of CML patients [7]. However, the persistence of residual tumor cells necessitates continued medication [25, 37]. Approximately 17% of patients display imatinib resistance within 5 years, highlighting the urgent need for innovative strategies to combat TKI resistance in CML treatment [38–41]. Previous studies have revealed metabolic shifts in CML resistance, including increased ROS production, imbalanced glutathione oxidation, and a heightened reliance on amino acid, fatty acid, and glutamine metabolism [42–46]. These studies provided a biological basis for metabolic intervention in IM-resistant CML management. Chiglitazar is a thiazolidinedione insulin sensitizer and has demonstrated potential in inhibiting leukemia stem cells [27]. However, its therapeutic efficacy in CML cells remains unexplored. We demonstrate in this study that Chi-mediated reprogram of glucose metabolism is capable of resensitizing IM-resistant CML cells for tranditional IM therapy.

**Fig. 9:**
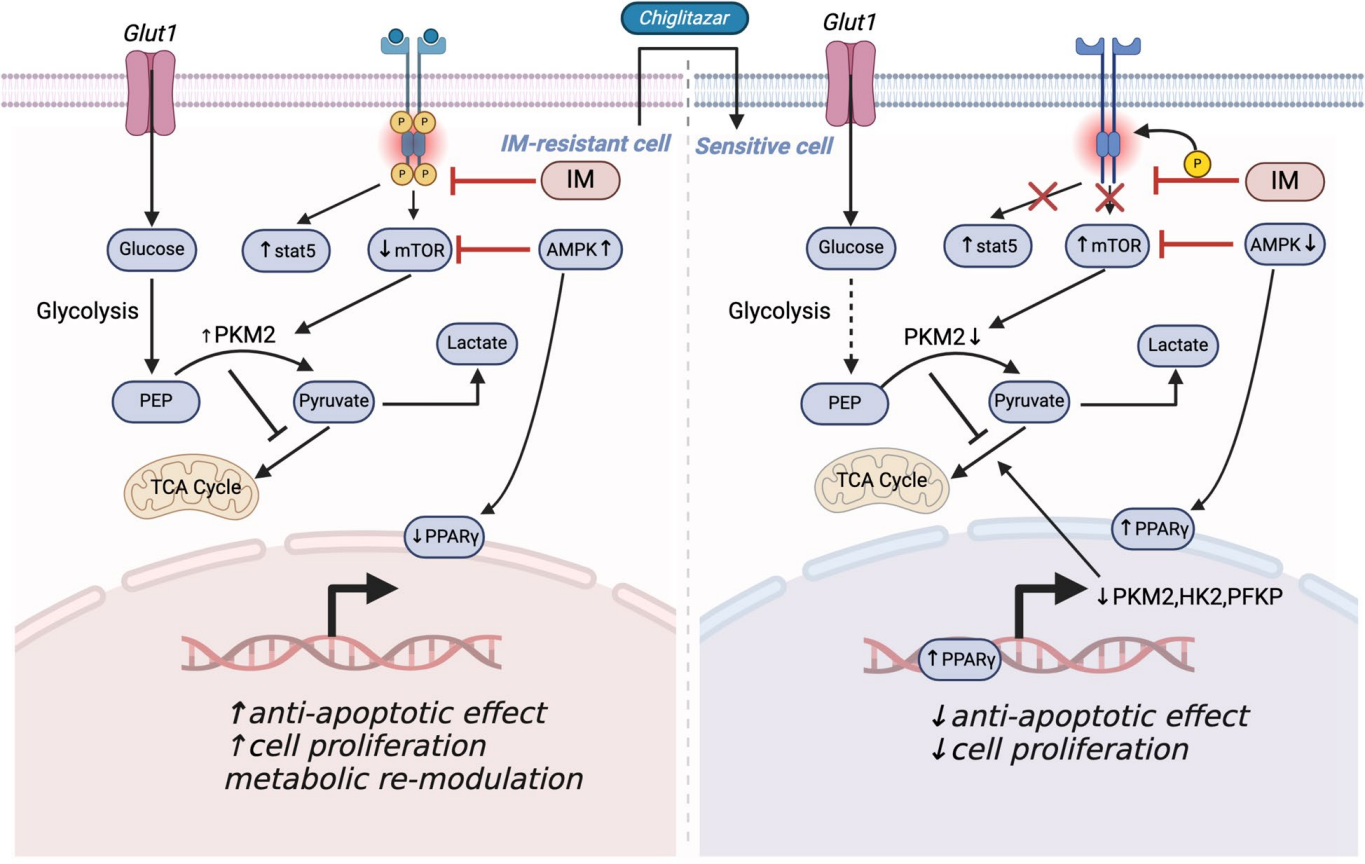
Schematic diagram of the function and mechanisms of Chiglitazar in CML cells. Chiglitazar activates PPARγ and therefore reduces the PKM2 expression. This molecule inhibits the lactate production of IM-resistant CML cells by inhibiting the mTOR signalling pathway. Chiglitazar causes mitochondrial dysfunction to activate the caspase pathway and then induces apoptosis in CML cells

In this study, we used transcriptomic sequencing to uncover important glucose metabolic differences between IM-resistant and sensitive cells, highlighting the discernible metabolic disparities. Metabolomic data also confirms the change in glucose metabolism. We then discovered that Chi could reduce glucose uptake and lactate production in sensitive and IM-resistant CML cells but didn’t induce CML cell apoptosis. It is consistent with the finding that CML cells with the *BCR::ABL* fusion gene tend to shift their energy metabolism toward active glycolysis. Our results also confirmed that IM-resistant CML cells, dependent on glycolysis, are more sensitive to glycolytic inhibition or glucose depletion (Fig. 2). TKI-resistant cells are reliant on glycolysis, particularly PKM2, which is a stress-associated rate-limiting enzyme [47, 48]. The combination of Chi and IM therapy inhibits CML cells effectively regardless of the presence or absence of *BCR::ABL* mutations T315I. Chi appears to exert cellular effects on CML cells primarily by inducing metabolic shifts, altering cell cycles, and contrasting the apoptosis typically induced by conventional chemotherapeutic agents [49, 50]. PPARγ is critical to glucose homeostasis and the adipogenic pathway, as it influences the activity of a network of genes involved in cell differentiation, proliferation, and apoptosis [51, 52]. Although PPARγ has been suggested as a tumor suppressor, there is conflicting evidence regarding its role in tumorigenesis, including cancers related to CML [25, 34, 53, 54]. Pyruvate kinase (PK) is a glycolysis regulator that orchestrates critical signaling pathways that promote cancer cell metabolism, proliferation, and migration [55–57]. Our study reveals that PPARγ activation reduces the phosphorylation of STAT5 but not its expression, which is essential for leukemia cell survival. Targeting PKM2 and STAT5 presents a potential drug target for eradicating IM-resistant CML, as supported by our findings of Chi and IM combination downregulating STAT5 via PKM2 (Fig. 9). Interestingly, our previous study proved that Chi increased PPARα expression, resulting in the inhibition of glucose metabolism and apoptosis of AML cells. This is different from the main regulatory mechanism in IM-resistant CML cells. Consequently, it is possible that the effect of Chi on IM-resistant CML cells observed in our study may also be mediated by multiple mechanisms. The mechanism of Chi to partially inhibit the Warburg Effect could be explained by its direct activation of PPARγ. Metabolic shift is only part of the Warburg Effect, it would be interesting to test the enzymes involved in pyruvate metabolism prioritize like: efficient ATP production via mitochondrial oxidative phosphorylation. The mechanism by which Chi inhibits the mitochondrial oxidative phosphorylation seems mainly due to the limitation of metabolic substrates like pyruvate. In the future, it would be interesting to measure the metabolic substrate changes that are caused by the combinationof Chi and IM in vitro and in vivo using [U-13C]-glucose.

In recent years, extensive research has been conducted regarding metabolism and imatinib drug resistance due to its significance in tumor growth, survival, and chemotherapy resistance [44, 58–60]. Despite insights from various perspectives, clinically applicable strategies to combat resistance remain elusive. Pioglitazone, a thiazolidinedione combined with imatinib, was effective in eliminating CML stem cells by inhibiting STAT5 through PPARγ activation [25]. However, its usage is limited by adverse effects such as heart failure and bladder cancer [26, 61]. This study demonstrates that Chiglitazar and imatinib synergistically inhibit CML-resistant cells’ glycolysis, which reduces the viability of IM-resistant cells and enhances their susceptibility to IM. Our findings present a promising therapeutic approach wherein the inhibition of the glycolytic pathway, coupled with TKI therapy, may overcome drug resistance. This warrants future clinical studies for an affordable intervention in TKI-resistant patients.

## Conclusions

Our study demonstrated the synergistic effect of Chi and IM on IM-sensitive and IM-resistant CML cells by activating the PPARγ and mTOR pathway, weakening the PKM2 and STAT5. This change thus causing mitochondrial dysfunction to activate the caspase pathway. Additionally, we demonstrated that Chi could improve the susceptibility of CML cells to IM. These experiments provide a mechanism basis for Chi as a solution for TKI-resistant patients.

## Supplementary Information

Additional file 1: Figure S1. (a–c) Typical flow cytometry diagrams of apoptosis level in K562, K562R, and Baf3-T315I cells treated with Chi-only, IM-only, and their combination for 48 h. (d) OCR level of K562 and K562R cells. (e) Glucose uptake and lactate production in K562 and K562R cells after Chi-only (20 μM), IM-only (5 μM), and their combination for 24 h. (f) Western blotting analysis of PPARγ protein after shPPARγ transfection. (g) Cell viability percentage of MHY1458 for 24, 48, and 72 h in K562R cells. (h) Western blotting analysis of mTOR expression and phosphorylation levels after MHY1458 treatment. (i) Typical flow cytometry diagrams of apoptosis level in K562R cells treated with IM-only, and MHY combination for 24, 48, 72 h. (j) Weight changes of CDX models after drug treatment with Chi-only, IM-only, and their combination for 16 days. Data are presented as mean ± SD. NS < 0.1234, *P < 0.03, **P < 0.0021, ***P < 0.0002, ****P < 0.0001.

Additional file 2: Table S1. Differential expressed gene list comparing K562R over K562 cells.

Additional file 3: Tables S2. Selected enriched biological process and pathways generated by DEGs of table S1.

Additional file 4: Table S3. 431 DEGs generated from venn diagram.

Additional file 5: Table S4. KEGG enrichment analysis of 431 DEGs in table S3.

## Data Availability

No datasets were generated or analysed during the current study.

## Abbreviations

CML: Chronic myeloid leukemia
TKI: Tyrosine kinase inhibitors
IM: Imatinib
Chi: Chiglitazar
CDX: Cell-derived xenograft
ECAR: Extracellular acidification rate
PPAR: Proliferator-activated receptor
PFK: Phosphofructokinase
CCK-8: Cell counting kit-8
CI: Combination index
