# Supplementary material for: Chiglitazar diminishes the warburg effect through PPARγ/mTOR/PKM2 and increases the sensitivity of imatinib in chronic myeloid leukemia
Source: Exp Hematol Oncol. 2024 Dec 18;13:121. doi: 10.1186/s40164-024-00589-1 (PMC11657277; doi:10.1186/s40164-024-00589-1)
Supplement: Supplementary file 1 — Additional file 1: Figure S1. (a–c) Typical flow cytometry diagrams of apoptosis level in K562, K562R, and Baf3-T315I cells treated with Chi-only, IM-only, and their combination for 48 h. (d) OCR level of K562 and K562R cells. (e) Glucose uptake and lactate production in K562 and K562R cells after Chi-only (20 μM), IM-only (5 μM), and their combination for 24 h. (f) Western blotting analysis of PPARγ protein after shPPARγ transfection. (g) Cell viability percentage of MHY1458 for 24, 48, and 72 h in K562R cells. (h) Western blotting analysis of mTOR expression and phosphorylation levels after MHY1458 treatment. (i) Typical flow cytometry diagrams of apoptosis level in K562R cells treated with IM-only, and MHY combination for 24, 48, 72 h. (j) Weight changes of CDX models after drug treatment with Chi-only, IM-only, and their combination for 16 days. Data are presented as mean ± SD. NS < 0.1234, *P < 0.03, **P < 0.0021, ***P < 0.0002, ****P < 0.0001. [file 40164_2024_589_MOESM1_ESM.pptx]

## Slide 1
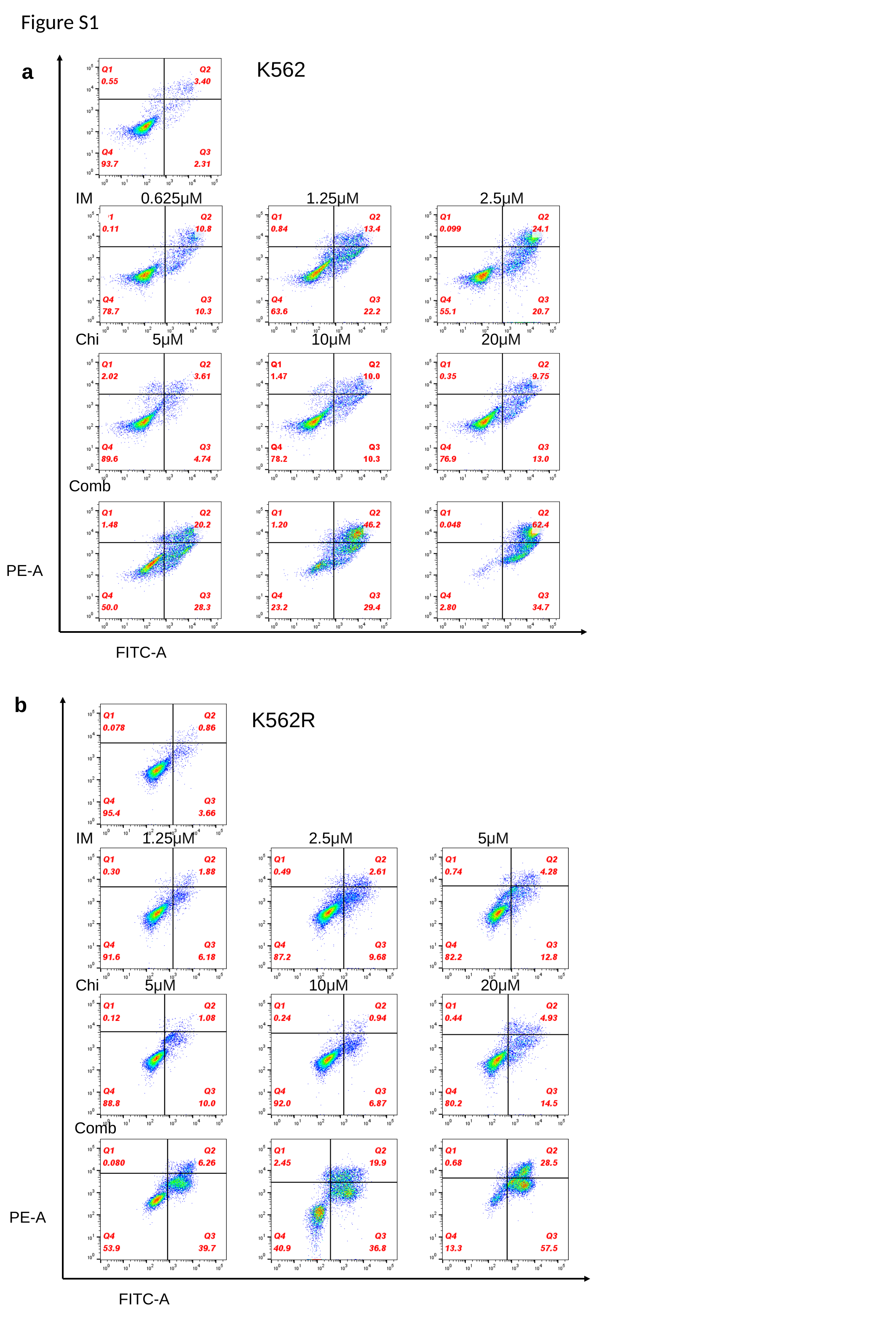

Figure S1
K562
a
IM
0.625μM
1.25μM
2.5μM
Chi
5μM
10μM
20μM
Comb
PE-A
FITC-A
b
K562R
IM
1.25μM
2.5μM
5μM
Chi
5μM
10μM
20μM
Comb
PE-A
FITC-A

## Slide 2
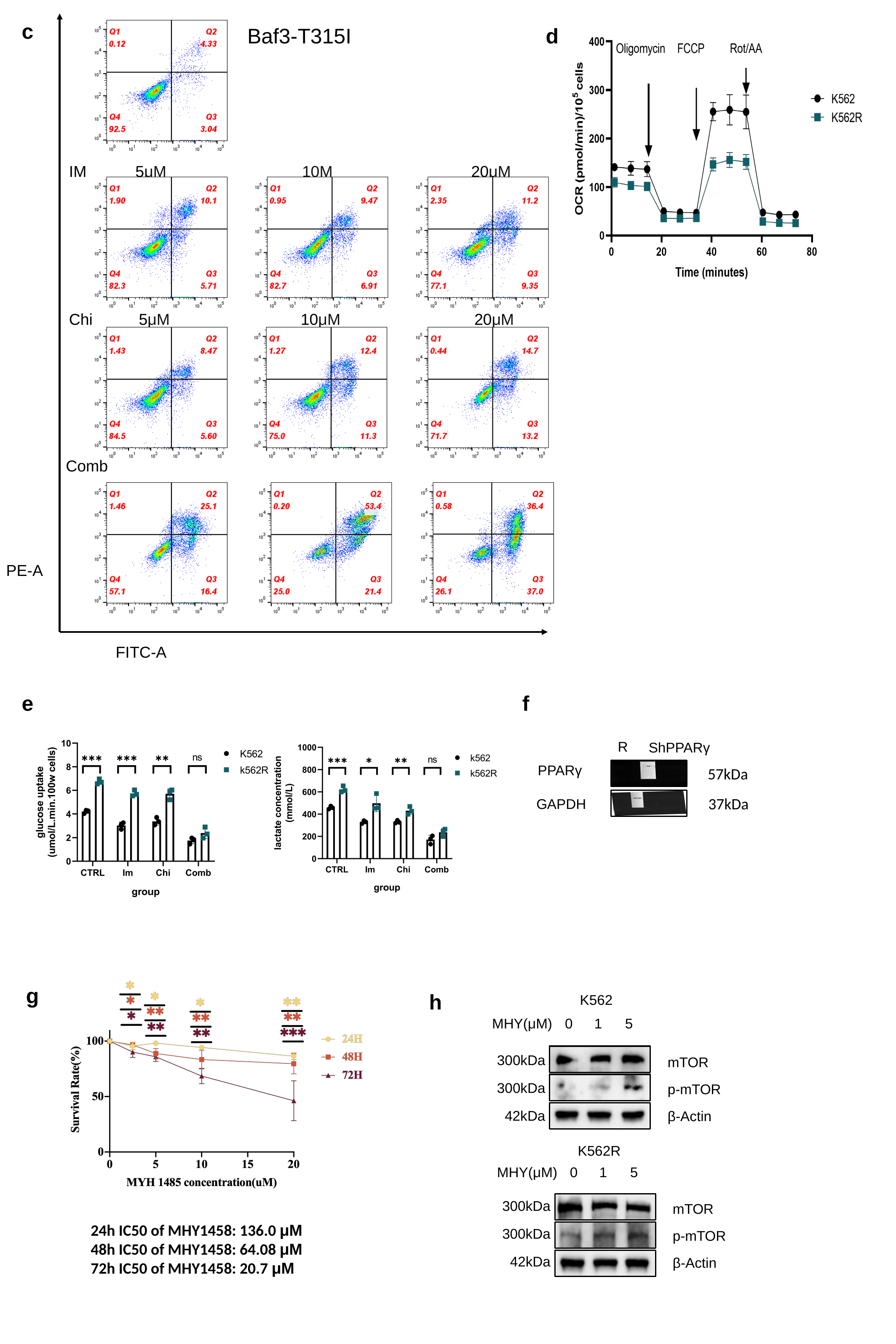

c
Baf3-T315I
d
IM
5μM
10M
20μM
Chi
5μM
10μM
20μM
Comb
PE-A
FITC-A
f
e
R
ShPPARγ
PPARγ
57kDa
37kDa
GAPDH
g
h
K562
| MHY(μM) | 0 | 1 | 5 |
| --- | --- | --- | --- |
300kDa
mTOR
300kDa
p-mTOR
42kDa
β-Actin
K562R
| MHY(μM) | 0 | 1 | 5 |
| --- | --- | --- | --- |
300kDa
mTOR
24h IC50 of MHY1458: 136.0 μM
48h IC50 of MHY1458: 64.08 μM
72h IC50 of MHY1458: 20.7 μM
300kDa
p-mTOR
42kDa
β-Actin

## Slide 3
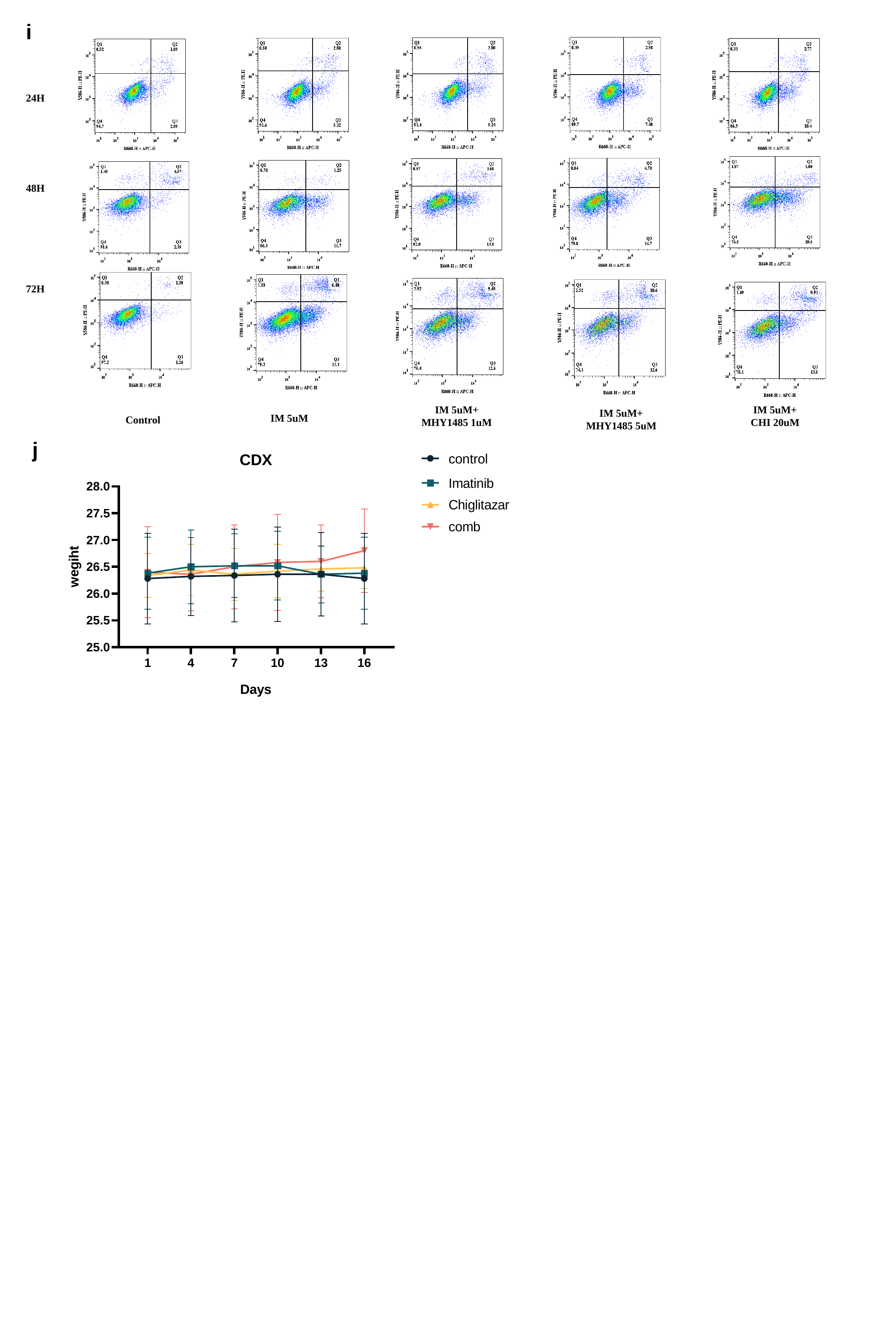

i
24H
48H
72H
IM 5uM+
MHY1485 1uM
IM 5uM+
CHI 20uM
IM 5uM+
MHY1485 5uM
IM 5uM
Control
j
